# Development and validation of a nomogram to predicting the efficacy of PD-1/PD-L1 inhibitors in patients with nasopharyngeal carcinoma

**DOI:** 10.1007/s12094-024-03504-6

**Published:** 2024-05-06

**Authors:** Yao Chen, Dubo Chen, Ruizhi Wang, Shuhua Xie, Xueping Wang, Hao Huang

**Affiliations:** 1 Department of Laboratory Medicine, The First Affiliated Hospital of Sun Yat-Sen University, Guangzhou, 510000 Guangdong China; 2 Department of Laboratory Medicine, Sun Yat-Sen University Cancer Center, Guangzhou, 510000 Guangdong China

**Keywords:** Immune checkpoint inhibitors, Nasopharyngeal cancer, Nomogram, Efficacy

## Abstract

**Purpose:**

With the treatment of nasopharyngeal carcinoma (NPC) by PD-1/PD-L1 inhibitors used widely in clinic, it becomes very necessary to anticipate whether patients would benefit from it. We aimed to develop a nomogram to evaluate the efficacy of anti-PD-1/PD-L1 in NPC patients.

**Methods:**

Totally 160 NPC patients were enrolled in the study. Patients were measured before the first PD-1/PD-L1 inhibitors treatment and after 8–12 weeks of immunotherapy by radiological examinations to estimate the effect. The least absolute shrinkage and selection operator (LASSO) logistic regression was used to screen hematological markers and establish a predictive model. The nomogram was internally validated by bootstrap resampling and externally validated. Performance of the model was evaluated using concordance index, calibration curve, decision curve analysis and receiver operation characteristic curve.

**Results:**

Patients involved were randomly split into training cohort ang validation cohort. Based on Lasso logistic regression, systemic immune-inflammation index (SII) and ALT to AST ratio (LSR) were selected to establish a predictive model. The C-index of training cohort and validating cohort was 0.745 and 0.760. The calibration curves and decision curves showed the precise predictive ability of this nomogram. The benefit of the model showed in decision curve was better than TNM stage. The area under the curve (AUC) value of training cohort and validation cohort was 0.745 and 0.878, respectively.

**Conclusion:**

The predictive model helped evaluating efficacy with high accuracy in NPC patients treated with PD-1/PD-L1 inhibitors.

## Background

Nasopharyngeal carcinoma (NPC) is an epithelial carcinoma occurring in the mucosa of the nasopharynx. It was regarded as a most common diagnosed head and neck malignant tumors. In 2020, there were 133,354 new cases of NPC diagnosed that year, according to the International Agency for Research on Cancer [1]. NPC shows distinct geographical distributing and is particularly common in East Asia, especially in southern China [2].

Traditionally, emphasized in guidelines, NPC is almost entirely relying on radiotherapy with concurrent chemotherapy to achieve disease control, particularly in the treatment of stage II to IVA disease [3, 4]. However, patients with recurrence and distant metastasis have limited benefit from radiotherapy. In the past few years, based on programmed death-1/programmed death ligand-1 (PD-1/PD-L1) blockade, immune checkpoint inhibitors (ICIs) enriched the thoughts for the treatment of patients with cancer, include NPC [5, 6]. PD-1/PD-L1 inhibitors in combination with gemcitabine and cisplatin are approved as first-line for the treatment of refractory recurrence and/or metastasis (R/M) NPC in 2021 in China. With the advances in immunotherapy, the incorporation of anti-PD-1/PD-L1 in the treatment of NPC has become a clinical focus [7, 8].

According to the International Union Against Cancer/American Joint Committee on Cancer (UICC/AJCC) tumor-node-metastasis (TNM) staging system, NPC is mainly classified by anatomy and EBV DNA [9]. Due to the limitations of current staging system in predicting prognosis or treatment effect, particularly in patients treated with ICIs, a lot of research to explore the other clinical factors and molecular biomarkers should be brought in the assessment system to predict prognosis. A multicenter cohort analysis suggested that a nomogram combined images from magnetic resonance imaging (MRI) features and clinical data is more accurate in predicting prognostic for patients with nasopharyngeal carcinoma [10]. Many studies have proved the prognostic value and potential clinical practice of molecular biomarkers, such as mRNAs, miRNAs, and DNA methylation.

Almost all the risk factors reported in precious studies have high requirements in equipment, which make it difficult and expensive to examine routinely. We aimed to set up a nomogram using common hematological indicators to predict the benefit of PD-1/PD-L1 inhibitors combinations in NPC patients.

## Method

### Patients selection

This study was retrospective and conducted at Sun Yat-sen University Cancer Center between January 1st, 2018 and December 31st, 2021. Adult patients with histopathological confirmation of NPC treated with anti-PD-1/PD-L1 combination therapy were included and randomly divided into training group and validation group. Patients with incomplete data or loss to follow-up were excluded. And patients received ICI therapy previously were throw away, included nivolumab, pembrolizumab or atezolizumab. Totally 112 patients were enrolled in training group and 48 patients in the same condition were included in validation group. The training group was used to set up a predictive model. The validation group was used to external examine the general applicability of the model.

### Data collection

Clinical data from electronic medical record system (EMR), experiment data from laboratory information system (LIS). Clinical characteristics contained age, gender, metastasis stage, clinical stage, TNM classification, anti-PD-1medication regime and therapeutic response. Laboratory examinations, including Blood Routine examination (WBC, ANC, ALC, MONO, EASO, BASO, NLR, LMR, SII, RBC, HGB, HCT, MCV, MCH, MCHC, PLT, NLR, PLR, PNI), biomedical tests (ALT, AST, LSR, ALP, GGT, TBA, TBIL, DBIL, IBIL, TP, ALB, GLB, A/G, UREA, CREA, UA, CK, LDH, GLU, TG, CHO, HDL-C, LDL-C, LHR, ApoA1, ApoB, ABR, CRP, SAA, CAR, SCR) and EBV DNA. Laboratory data was collected prior to the initiation of anti-PD-1/PD-L1 treatment.

ANC is absolute neutrophil count. ALC is absolute lymphocyte count. NLR is calculated by the ratio of neutrophil to lymphocyte. LMR is calculated by the ratio of lymphocyte-monocyte. PLR is calculated by the ratio of platelet to lymphocyte. SII is PLT multiplied by NLR. PNI is calculated by ALB and five-fold of ALC. LSR is calculated by ALT to AST ratio. LHR is calculated by HDL-C to LDL-C ratio. ABR is calculated by ApoA1 to ApoB ratio. CAR is calculated by CRP to ALB ratio. SCR is calculated by SAA to CRP ratio.

Routine Blood Test was determined by Sysmex XN 9000, biochemical test was determined by Roches 8000 702, EBV DNA was examined by Roches Combas 480.

### Follow-up

ICI therapies in the study were defined as the use of one of Atezolizumab, Nivolumab, Pembrolizumab, Camrelizumab, Sintilimab, Toripalimab or Tislelizumab. In the training and validation cohorts, the therapeutic benefits of immunotherapy at 8–12 weeks were assessed by radiography according to solid tumor response assessment criteria (RECIST, version 1.1), which classified as partial response (PR), stable disease (SD), and progressive disease (PD). In this study, both partial response and stable disease are seemed disease control (DC).

### Statistical analysis

Lasso logistic regression was used to select significant markers in the training cohort. As the penalty coefficient λ increases, LASSO contracts all regression coefficients towards zero. The optimal values of the parameter λ was determined by ten-fold cross-validation with the 1 standard error (1-SE) of the minimum criteria (the 1-SE criteria), and the final value of λ produces the minimum cross-validation error. Variables corresponding to the coefficients with the estimated value of 0 in hypothetical λ are eliminated. By this means, the independent variables closely related to the dependent variable are picked out to build a model [11].

Continuous variables are represented by median and IQR, regardless of normal or non-normal distributions. Categorical variables are expressed in frequency and percentages. Kaplan–Meier method estimates the response. A predictive model of tumor response was established on account of the markers selected in the LASSO. The nomogram was developed for predicting the tumor response of NPC patients treated by anti-PD-1/PD-L1 therapy. Harrell’s C-index was evaluated on a range between 0 and 1 to quantify the discriminating performance of the nomogram. The C-index value above 0.75 was considered to indicate a good degree of consistency. The decision curve was plotted for the model of nomogram. Calibration curves that are close to ideal are considered to have accurate predictive power. With the area under curve (AUC), receiver operation characteristic (ROC) curve analyzes the prediction value of the model.

A test P-value < 0.05 indicated a statistically significant difference. Data were analyzed with the IBM SPSS Statistics for Windows (version 24.0) and R software (version 3.1.4; http://www.Rproject.org).

## Results

### Patient characteristics

A total of 160 subjects from Sun Yat-sen University Cancer Center recieving PD-1/PD-L1 antibody were recruited in this study: 112 in the training cohort and 48 in the validation cohort. Patients in this study ranged between 28 and 72 years old. The majority of patients in the training cohort were male (69.64%) and the average age is 47.4(41, 56.3) years old. The average age of patients in the validation cohort is also 46.9(40, 55) years old and 66.67% (32/48) were male.

In this study, all patients used PD-1/PD-L1 inhibitors regularly. Among the 160 patients, 103 experienced lymph node metastases, while 57 did not have a lymph node metastasis. Distant metastasis occurred in 64 cases, while 96 have no distant metastasis. The response of NPC patients to PD-1/PD-L1 inhibitor treatment was initially assessed at 8–12 weeks and constantly updated. All patients were followed more than 12 months. Patients were randomly divided into two groups at a ratio of 7:3.

Up to December 31, 2021, there were 91 (81.25%) patients had their disease under control in training cohort and 39 (81.25%) in validating cohort. Respectively, 21(18.75%) and 9(18.75%) patients’ progressive disease in the training and validation cohorts. Overall, 130 of 160 patients (81.25%) achieved disease control.

No significant differences were observed between the two groups. The demographic and clinical characteristics of patients in the training and validation cohorts are listed in Table 1.

**Table 1:** Demographic and clinical characteristics of NPC patients in the training and validation cohorts

Characteristic	All (n = 160)	Training cohort (n = 112)	Validation cohort (n = 48)	P
Sex, n(%)				0.911
Male	111(68.38%)	78(69.64%)	33(68.75%)	
Female	49(30.63%)	34(30.36%)	15(31.25%)	
Age(year)	47.2(41,54)	47.4(41,56.3)	46.9(40,55)	0.788
Clinical stage				0.871
II	5(3.12%)	3(2.68%)	2(4.12%)	
II	39(24.38%)	27(24.11%)	12(25.00%)	
IV	116(72.50%)	82(73.21%)	34(70.83%)	
Tumor stage				0.072
T0–2	27(16.88%)	15(13.39%)	12(25.00%)	
T3–4	133(83.12%)	97(86.61%)	36(75.00%)	
Node stage				0.692
N0–1	57(35.63%)	41(36.61%)	16(33.33%)	
N2–3	103(64.37%)	71(63.40%)	32(66.67%)	
Metastasis stage				0.324
M0	96(60.00%)	70(62.50%)	26(54.17%)	
M1	64(40.00%)	42(37.50%)	22(45.83%)	
Outcome				1
DC	130(81.25%)	91(81.25%)	39(81.25%)	
PD	30(18.75%)	21(18.75%)	9(18.75%)	

### Independent prognostic factors selection

**Fig.1:**
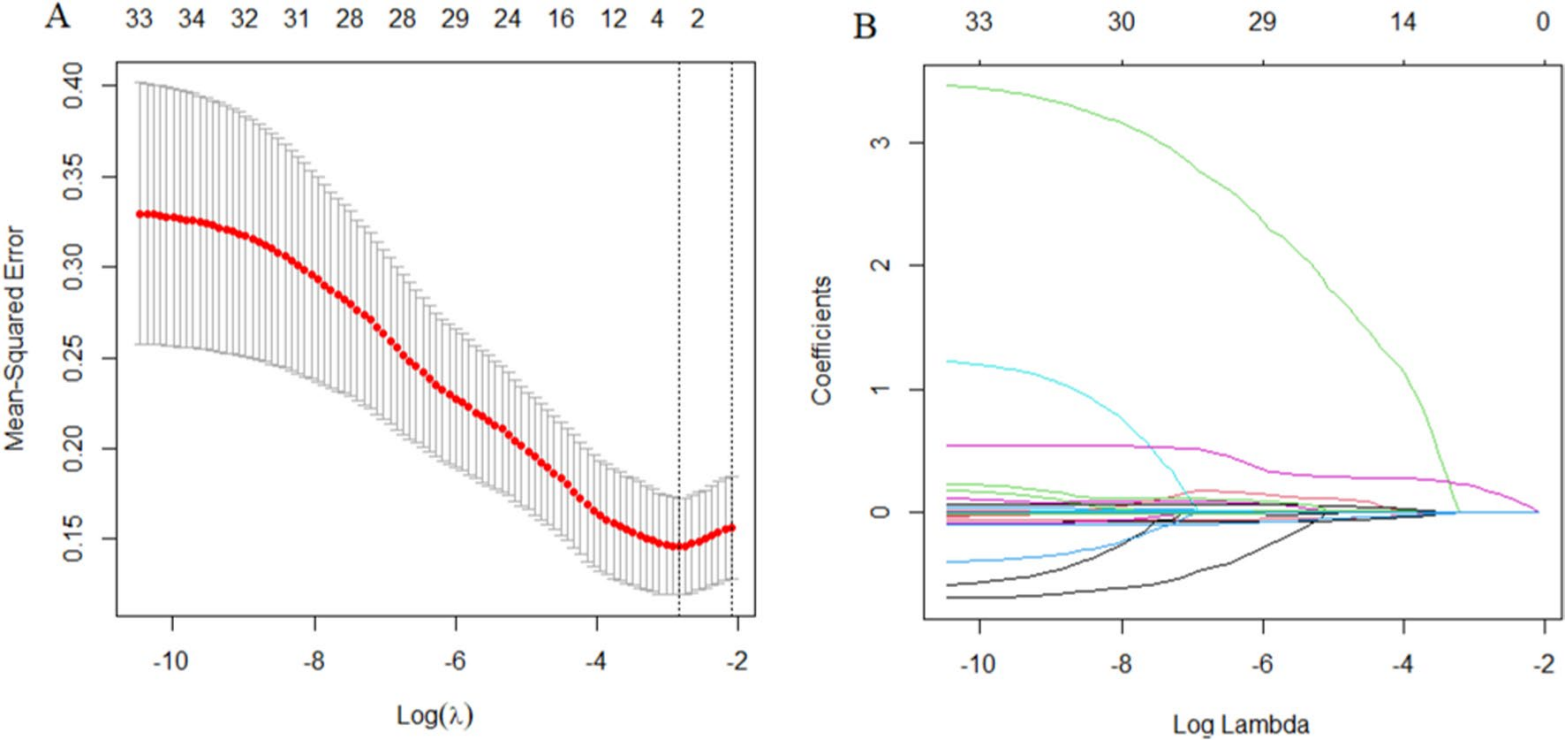
Prediction of the efficacy of NPC patients treated with PD-1 inhibitors based on prognostic factors. **A** Parameter (λ) selection in the Lasso model used tenfold cross-validation based on the minimum criteria. **B** Changes in 33 marker coefficients with the penalty parameter (λ)

LASSO regression can purify the most critical predictors from the virgin data set and is particularly suitable for working with high-dimensional data. LASSO logistic regression model could establish a risk score model, which simplified 50 variables into 2 potential predictors. The distribution of coefficients is shown in Fig. 1A, and the cross-validated error plot is shown in Fig. 1B. ALT to AST ratio (LSR) and systemic immune-inflammation index (SII) were determined by the training set (n = 112), these indicators were considered to predict the effect of anti-PD-1/PD-L1 therapy.

### Predictive nomogram

**Fig. 2:**
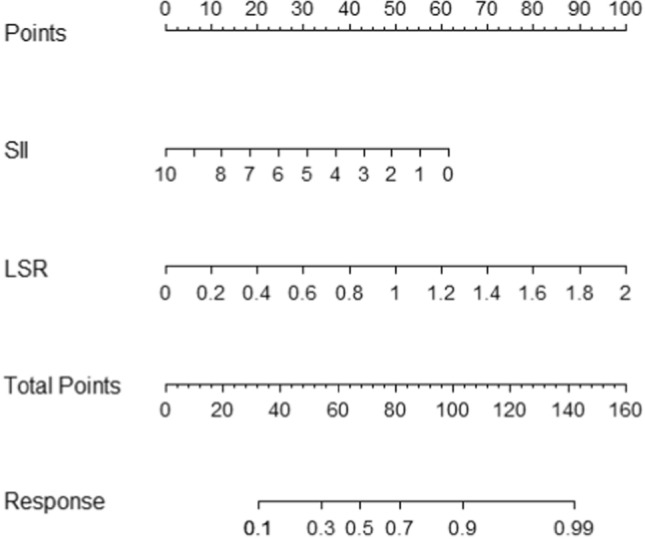
Nomogram for the prediction of disease control with Lasso selected factors

Based on the LASSO regression analysis, all independent predictive factors were integrated into a predictive model for visualization in of a nomogram form (Fig. 2). Each predictor corresponds to a score in the nomogram, ranging from 0 (lowest risk) to 100 (highest risk). In the nomogram, LSR was the most significant risk factors, while SII showed a noteworthy impact on treatment outcomes. By adding total scores together and drawing a vertical line from the position of total points, the estimated probability of tumor response of NPC patients could be obtained.

### Clinical utility of the nomogram

**Fig. 3:**
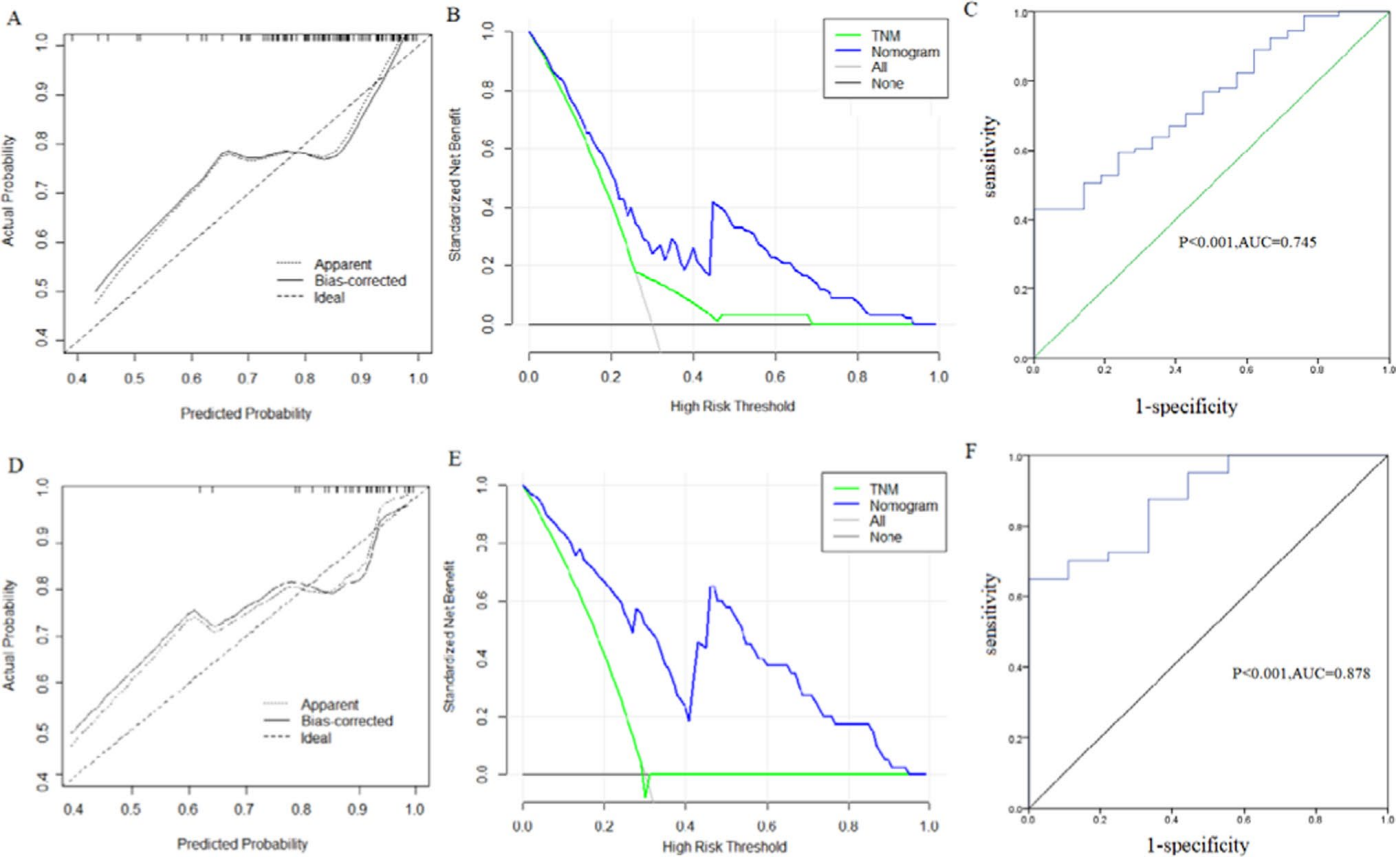
**A** Calibration plots of training cohort for predicting treatment effect. X-axis: bootstrap-predicted; y-axis: actual outcome. **B** Decision curve analysis (DCA) of the nomogram for predicting treatment effect in training cohort. X-axis: cut-off probability; y-axis: net benefit. **C** Receiver operating characteristic (ROC) curve of training cohort with area under the curve values.** D** Calibration plots of validation cohort for predicting treatment effect. **E** Decision curve analysis (DCA) of the nomogram for predicting treatment effect in validation cohort. **F** Receiver operating characteristic (ROC) curve of validation cohort with area under the curve values

To validate the clinical validity of the model and demonstrate the universality of nomogram, we validated our model with a validation cohort. C-index analysis showed that the C-index of training cohort and validity cohort provided by nomogram were 0.745 and 0.760, respectively. The calibration curves of training cohort (Fig. 3A) and validating cohort (Fig. 3D) showed good agreement with the ideal line of 45-degree. The nomogram has good accuracy in predicting the prognosis of NPC patients. Decision curve analysis of training cohort (Fig. 3B) and validation cohort (Fig. 3E) also suggested the potential clinical effects. During the curves, the new model showed the greatest net benefits in predict therapeutic benefits. And the prediction value of both training cohort (Fig. 3C) and validation cohort (Fig. 3F) of the model were proved by receiver operation characteristic curve with the area under curve. The area under the curve (AUC) in training cohort are 0.745 (P < 0.001) and that of the validation cohort was 0.878 (P < 0.001). The ROC curve displayed the extraordinary sensitivity and specificity of the model.

## Discussion

Despite radiotherapy with concurrent chemotherapy is still standard treatment, increasingly more NPC patients benefit from PD-1/PD-L1 inhibitors. However, the success of PD-1/PD-L1 inhibitors in disease control for NPC accompanied by uncertainty. Not all patients are fortunate enough to make good changes in disease development.

In the present study, the clinical characteristics and risk factors of NPC patients treated with anti-PD-1/PD-L1 were described, and the prognostic model of NPC patients was constructed to provide a theoretical basis for predicting the curative effect. In this study, two factors significantly associated with the effect, which include LSR and SII. These predictors used in nomogram can be easily accessed from clinical information and laboratory information systems and are feasible to apply in clinical practice.

Tumor microenvironment (TME) is a complex and rich multicellular system which contains tumor cells, lymphocytes (T-cells, B-cells, NK cells), myeloid populations [myeloid-derived suppressor cells (MDSCs), tumor-associated macrophages (TAMs), dendritic cells, tumor-associated neutrophils (TANs)], stromal cells [such as carcinoma-associated fibroblasts (CAFs)], lymphatic vascular networks, vascular system and extracellular matrix components(cytokines, growth factor, chemokins) [12, 13]. It is widely recognized that TME play an important roll in dynamically regulating cancer progression and influencing treatment outcome [14].

It is generally believed that inflammation is crucial in cancer and involved in various stage of cancer development, including initiation, progression, malignant transformation, metastasis, and moreover [15, 16]. In confirmed cancers, there is oceans of evidence that local immune responses and systemic inflammation play a role in building pro-tumor microenvironments in tumor development [17]. Changes in blood-based biomarkers may reflect the development of the disease in patients treated with immune checkpoint inhibitors (ICIs) [18].

SII is a continuous parameter calculated by three types of white cells (peripheral neutrophils, platelets, and lymphocytes) which was studied in various cancers, such as hepatocellular carcinoma and renal cell cancer [19, 20]. Zeng et al. conducted a retrospective study of 559 patients with NPC and 500 patients with chronic rhinitis, and found that the inflammatory parameters, including SII, were significantly higher in NPC patients before treatment than in patients with chronic rhinitis [17]. Another study conducted by Wang et al. proposed that the level of SII during treatment is a promising indicator of survival in NPC patients. The inflammation index could be used as independent prognostic factors for cancers, and they have higher prognostic value than only white blood cells [21]. In our study, high SII is associated with poor efficacy in NPC patients treated with PD-1/PD-L1 inhibitors.

Typically, the level of serum ALT and AST were measured to reviewing liver function, and LSR is also used as an indicator of liver function [22]. In recent decade, some researchers pointed it may have some associations with tumors. One study reported that AST/ALT ratio was associated with the efficacy in cancer patients by predicting chemotherapy-induced thrombocytopenia risk [23, 24]. Another study showed that baseline LSR is associated with the prognosis of patients with gastric adenocarcinoma [24]. What’s more, GGT and AST/ALT are reported to be independent factors that relate to the overall survival rate of esophageal squamous cell carcinoma [25].

In our study, we find that the baseline LSR is connected to the treatment effect of PD-1/PD-L1 inhibitors in NPC patients. Patients with higher baseline LSR levels are more likely to have a better tumor response. Conducted in the same institution, another study proved that the efficacy of anti-PD-1 in NPC patients may involve the dynamic changes of LSR [26]. Although there are some evidence, the use of LSR to evaluate the benefit of NPC patients treated with anti-PD-1/PD-L1 needs to be proofed in more studies.

This study was limited in some ways. Firstly, the number of NPC patients is large in our hospital, but the efficacy evaluation requires a long follow-up period so that the subjects involved is limited. Second, it was a retrospective study, which may have affected the ability of the analysis to construct the model. Further prospective studies are needed to validate the model.

This study have some limitations. Firstly, the volume of patients involved is limited. Secondly, it was a retrospective single center study. This may have influenced the power of the analysis to build model. Further prospective studies are needed to validate the utility of the nomogram in clinical with larger and more diverse patient cohort.

To our knowledge, few studies in China concentrated on efficacy evaluation in NPC patients treated with PD-1/PD-L1 antibodies and the performance is not so good [27]. This study established a model for predicting tumor response of NPC patients treated with PD-1/PD-L1 inhibitors for the first time, providing a beneficial basis for the treatment of NPC patients. Physicians can quickly predict the therapeutic effect of ICI treatment in NPC patients and propose therapeutic plans based on the therapeutic evaluation. Additional studies would be required to explore the mechanisms and predictors of non-sense.

## Conclusion

In summary, our study focus on NPC patients treated with PD-1/PD-L1 inhibitors and build a new nomogram to evaluate the efficacy of these patients. The nomogram may help clinicians to simply estimate treatment efficacy of NPC patients treated with PD-1/PD-L1 inhibitors in the early stage of treatment.

## Data Availability

The original contributions presented in the study are included in the article. Further inquiries can be directed to the corresponding author.
